# A Rare Case of Gastric Fundal Vascular Ectasia: A Unique Presentation and Diagnostic Challenge

**DOI:** 10.7759/cureus.82296

**Published:** 2025-04-15

**Authors:** Shirisha Saripalli, Hiba Suliman, Hafiz Haseeb, Faisal Nawaz, Huda Khan

**Affiliations:** 1 Medicine, Grange University Hospital, Cwmbran, GBR; 2 Gastroenterology and Hepatology, Royal Albert Edward Hospital, Wigan, GBR; 3 Gastroenterology, Grange University Hospital, Cwmbran, GBR; 4 Gastroenterology and Hepatology, Grange University Hospital, Cwmbran, GBR; 5 Gastroenterology, Bakhtawar Amin Medical and Dental College, Multan, PAK

**Keywords:** argan plasma coagulation, bleeding gastric varices, plasma coagulation, pulsed argon, s: acute variceal upper gastrointestinal bleed, vascular ectasia

## Abstract

A 75-year-old male patient was admitted with a two-week history of melena on a background of psoriatic arthritis and chronic iron-deficiency anaemia. Initial bloods confirmed acute-on-chronic anaemia with evidence of ongoing bleeding as the hemoglobin consistently dropped to 77g/L from 84g/L. After resuscitation which included giving a unit of blood transfusion, an urgent endoscopy revealed moderate gastric fundal vascular ectasia (GFVE) with active bleeding from three points and mild gastric antral vascular ectasia with no bleeding. Pulsed Argon-Plasma coagulation was successfully applied during endoscopy immediately, achieving haemostasis. Small Grade-2 oesophageal varices without bleeding were also noted. Subsequent imaging confirmed new liver cirrhosis with CHILD-PUGH scoring of 6 falling into category A, which was under investigation as outpatient. The patient’s haemoglobin stabilized post-endotherapy with significant symptoms improvement and he was also given intravenous iron infusion. No further gastrointestinal bleeding event was noted until the writing of this article.

## Introduction

Gastric vascular ectasia (GVE) is an extremely uncommon condition that contributes to gastrointestinal bleeding in 4 out of 100 cases. While gastric vascular ectasia predominantly affects the antrum, involvement of other parts of the gastrointestinal tract, such as the fundus, duodenum, jejunum, and rectum, has been rarely reported [1]. Although GVE is often asymptomatic, it can result in anaemia due to ongoing blood loss from the affected vessels [2]. It predominantly affects elderly females (approximately 89%), with the most common presenting symptom being anaemia caused by prolonged bleeding. GVE has been associated with various underlying conditions, including liver cirrhosis (in about 30% of cases), autoimmune connective tissue disorders, Raynaud's phenomenon, and, less frequently, chronic renal failure, bone marrow transplantation, and cardiac disease [3].

The pathogenesis of GVE remains poorly understood, although several hypotheses have been proposed [4]. The diagnosis is primarily based on endoscopic findings, which may include a characteristic radial pattern of stripes originating from the pylorus, often referred to as a "watermelon stomach". This pattern is most commonly seen in non-cirrhotic patients. Alternatively, a diffuse "honeycomb" pattern may be observed, which is more typical in patients with liver failure [5]. Histopathological examination typically reveals vascular ectasia of mucosal capillaries, focal thrombosis, spindle cell proliferation, and fibrohyalinosis-homogeneous material surrounding the ectatic capillaries in the lamina propria [2]. The main differential diagnosis to consider is portal hypertensive gastropathy [3].

Management strategies for GVE include blood transfusions to address anaemia. Surgical options such as antrectomy, as well as pharmacologic therapies including hormonal treatment and octreotide, may be used to control bleeding [4]. Endoscopic interventions, such as laser photoablation and Pulsed Argon-Plasma coagulation, are also commonly employed to manage bleeding episodes.

The aim of this report is to present a case with such a finding and to create educational awareness of this diagnosis at an uncommon location.

## Case presentation

**Figure 1:**
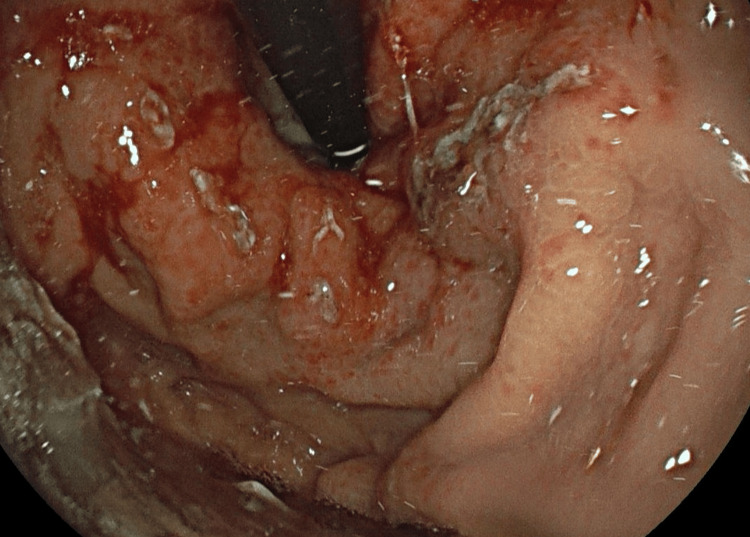
Gastric Fundal Ectasia - Endoscopic image showing vascular ectasia confined to the gastric fundus, with erythematous mucosa and prominent, dilated submucosal vessels.

**Figure 2:**
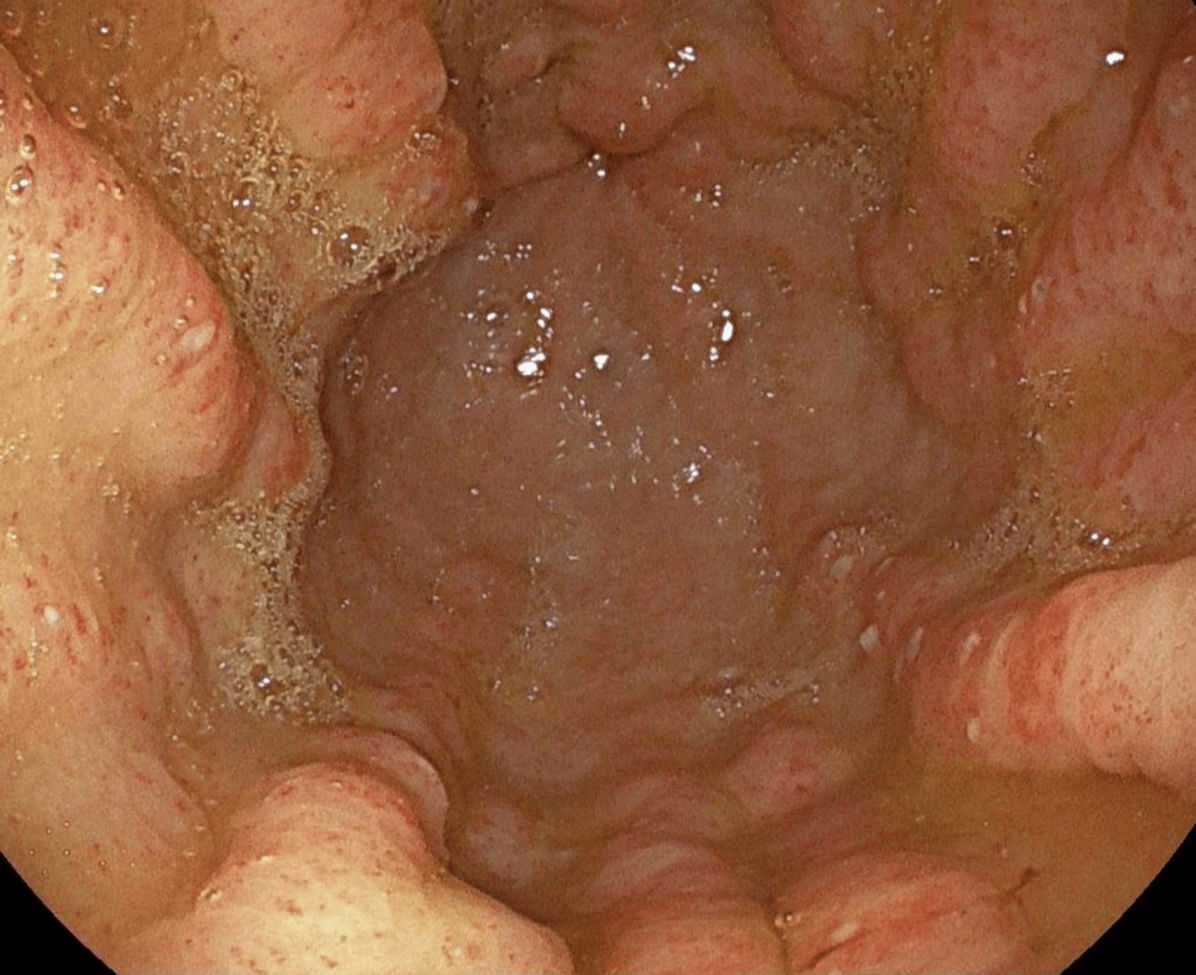
Gastric Fundal and Antral Ectasia - Endoscopic view demonstrating diffuse vascular ectasia involving both the gastric fundus and antrum, with mucosal erythema and prominent vascular patterns.

**Figure 3:**
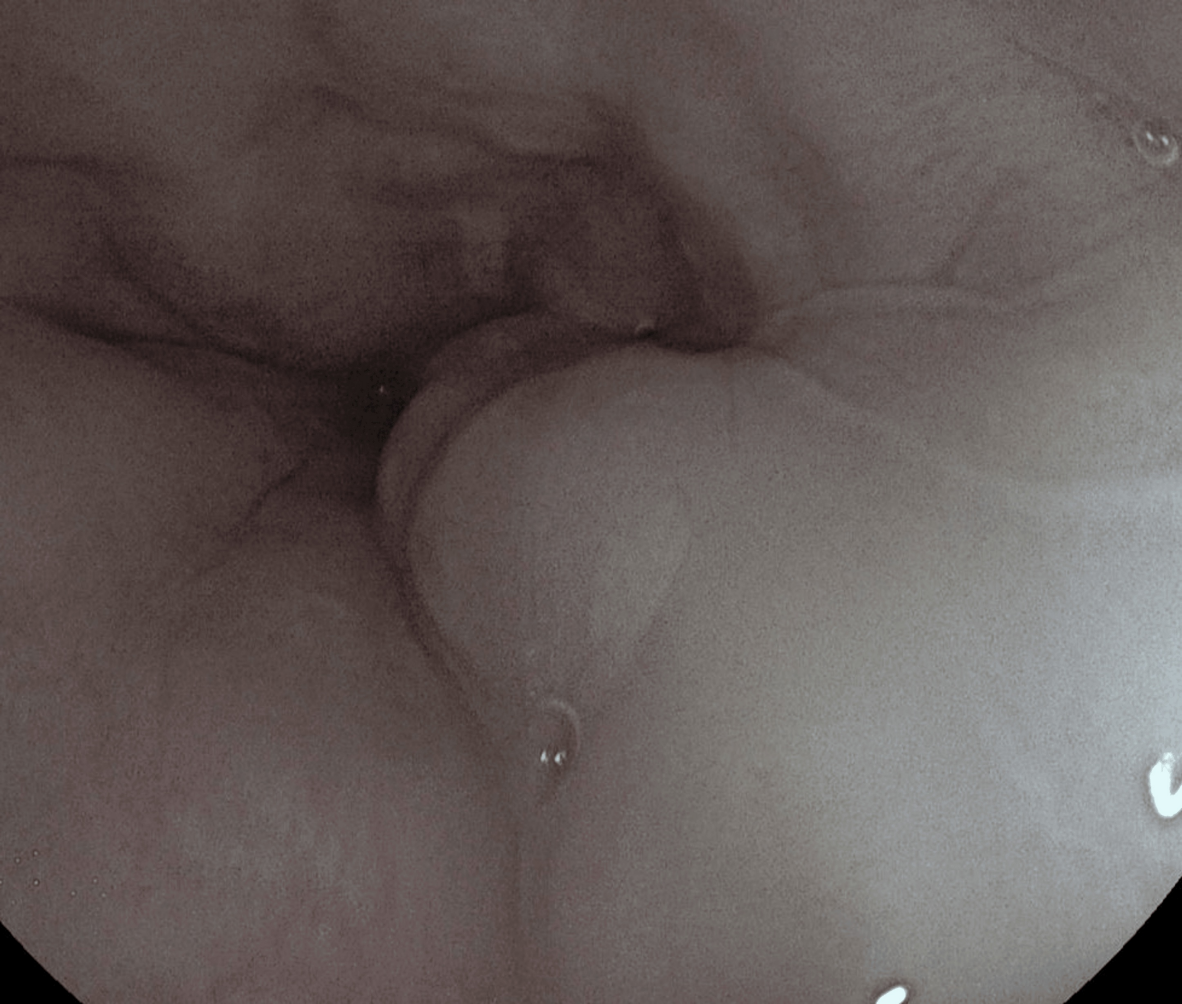
Oesophageal varices - Endoscopic image showing large oesophageal varices as serpiginous, bluish submucosal elevations in the distal oesophagus, consistent with portal hypertension.

**Figure 4:**
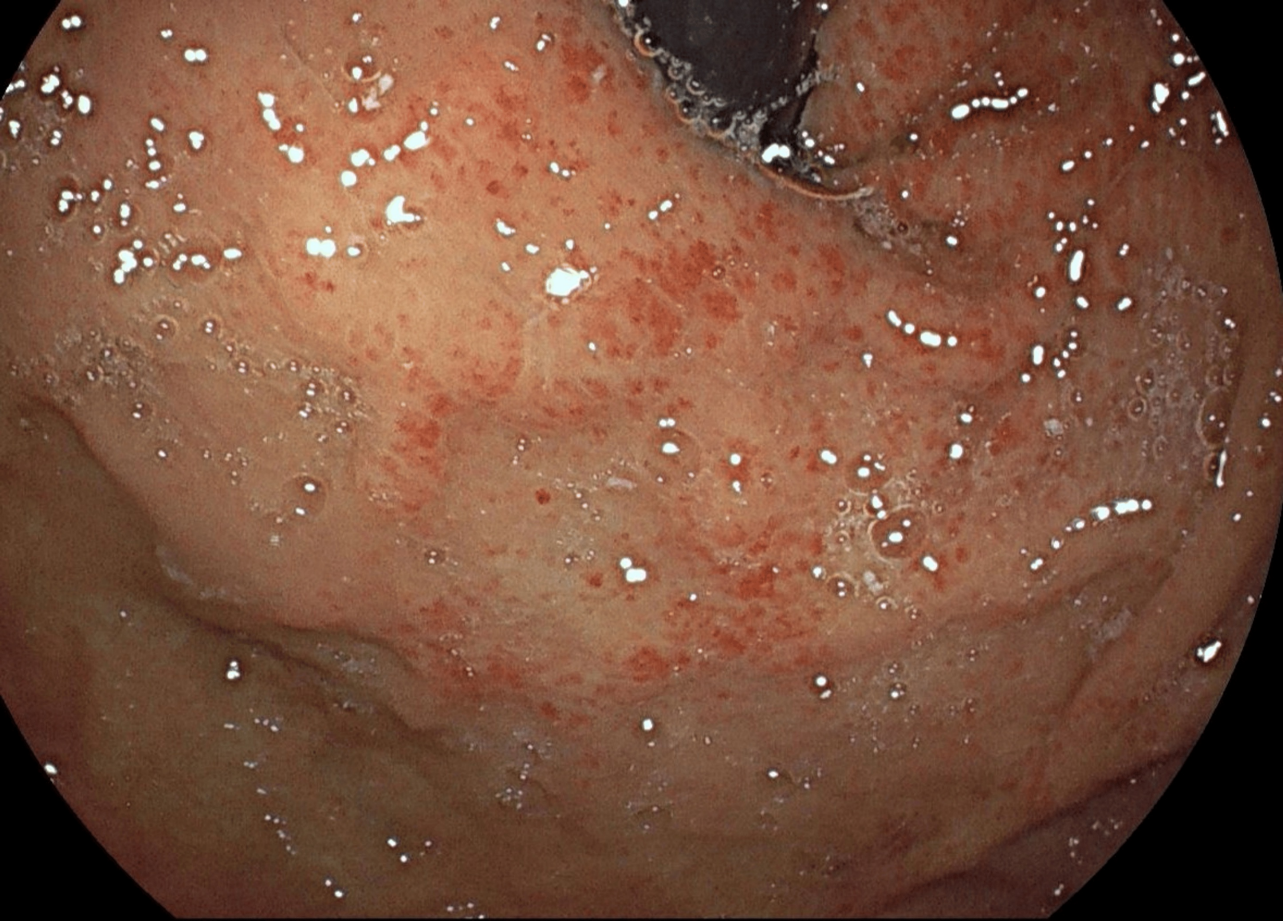
Post APC repeat scope - Follow-up endoscopic image post-argon plasma coagulation (APC) therapy, showing scattered erythema and coagulation marks in the gastric mucosa indicating treatment response.

A 75-year-old male patient was admitted with a two-week history of melena, and he has a medical history of psoriatic arthritis and chronic iron-deficiency anaemia. Initial bloods confirmed acute-on-chronic anaemia with evidence of ongoing bleeding. After resuscitation, an urgent endoscopy revealed moderate gastric fundal vascular ectasia (GFVE), as illustrated in Figure 1 with active bleeding from three points and mild gastric antral vascular ectasia, which is shown in Figure 2 with no bleeding. Small Grade-2 oesophageal varices as seen in Figure 3 without bleeding were also noted. Pulsed Argon-Plasma coagulation was successfully applied and this has been illustrated in Figure 4, achieving haemostasis. Subsequent imaging confirmed new liver cirrhosis, which was under investigation as outpatient. The patient’s haemoglobin stabilized post-endotherapy with significant symptoms improvement.

## Discussion

GVE is a condition commonly seen in the elderly and is often an underdiagnosed source of chronic blood loss, leading to refractory anaemia. It can also present as a severe upper gastrointestinal bleed, requiring immediate management [3]. The exact pathophysiology of GVE remains unclear, though several theories have been proposed, including achlorhydria, hypergastrinemia, and low pepsinogen levels [4]. The underlying causes of the histologic changes, particularly fibromuscular growth in the lamina propria and vascular dilation with thrombosis, are still unknown [5]. Elevated levels of vasodilatory hormones like gastrin and prostaglandin E2 have been observed in GFVE patients, and it is suggested that impaired liver function may contribute to a buildup of these hormones, playing a role in the disease's pathogenesis. Another common theory highlights mechanical stress, proposing that abnormal peristaltic waves in the gastric antrum create pressure that causes mucosal prolapse through the pyloric ring, triggering long-term inflammation [5,6].

Diagnosis is primarily based on the characteristic endoscopic appearance, although biopsies may be necessary in uncertain cases. The main treatment is endoscopic argon plasma coagulation (APC), while endoscopic band ligation may be used for large, localized vascular lesions. In refractory cases, partial gastrectomy may be considered as a last resort [5].

As mentioned in the literature, GVE is typically found in the antrum, and female preponderance is higher (5:1) in especially elderly patients, but in this case, it was a male patient with a fundal presentation more commonly referred to as GFVE [4]. The condition may present with a spectrum ranging from chronic blood loss to acute gastrointestinal hemorrhage. Although acute bleeding is uncommon, it was noted in this case, where the patient exhibited haematemesis [6].

Two characteristic endoscopic patterns have been described: red punctate lesions, often seen in patients with liver cirrhosis, and red lesions arranged in stripes, typically observed in females with connective tissue disorders. However, in this patient, despite having liver cirrhosis, no red sign was observed on Endoscopy.

Due to its rare presentation, some cases have been misdiagnosed initially, possibly because of similar findings, such as blood flow patterns or the presence of other gastric conditions like gastritis and portal hypertensive gastropathy, that usually affects the proximal part of the stomach [5]. Treatment generally involves initial resuscitation and symptomatic care, with medical therapy aimed at reducing portal pressures, although its effectiveness is limited [1]. Endoscopy is typically the preferred first-line therapy, which was the approach taken in this case, without the use of medical treatment. In refractory cases, antrectomy has shown some benefit, though it carries a significant risk of mortality [3].

## Conclusions

GVE is a rare yet significant cause of upper gastrointestinal bleeding that can present in different locations, the most common one being the antrum, hence, called as gastric antral vascular ectasia (GAVE), but in the case presented above, it was in the fundus, therefore, called as GFVE. Its diagnosis can sometimes be missed during upper GI endoscopy, so it should always be considered, particularly in cases of obscure GI bleeding. The primary treatment option is endoscopic therapy with argon plasma coagulation.
